# Upregulation of the transcription factor TFAP2D is associated with aggressive tumor phenotype in prostate cancer lacking the *TMPRSS2:ERG* fusion

**DOI:** 10.1186/s10020-020-00148-4

**Published:** 2020-03-06

**Authors:** Christoph Fraune, Luisa Harms, Franziska Büscheck, Doris Höflmayer, Maria Christina Tsourlakis, Till S. Clauditz, Ronald Simon, Katharina Möller, Andreas M. Luebke, Christina Möller-Koop, Stefan Steurer, Claudia Hube-Magg, Guido Sauter, Sören Weidemann, Patrick Lebok, David Dum, Simon Kind, Sarah Minner, Jakob R. Izbicki, Thorsten Schlomm, Hartwig Huland, Hans Heinzer, Eike Burandt, Alexander Haese, Markus Graefen, Cornelia Schroeder

**Affiliations:** 1 Institute of Pathology, University Medical Center Hamburg-Eppendorf, Martinistr 52, 20246 Hamburg, Germany; 2 General, Visceral and Thoracic Surgery Department and Clinic, University Medical Center Hamburg-Eppendorf, Hamburg, Germany; 3 Department of Urology, Charité - Universitätsmedizin Berlin, Berlin, Germany; 4 Martini-Clinic, Prostate Cancer Center, University Medical Center Hamburg-Eppendorf, Hamburg, Germany

**Keywords:** TFAP2D, prostate cancer, genomic instability, prognosis, immunohistochemistry, tissue micro array

## Abstract

**Background:**

TFAP2D is a transcription factor important for modulating gene expression in embryogenesis. Its expression and prognostic role in prostate cancer has not been evaluated.

**Methods:**

Therefore, a tissue microarray containing 17,747 prostate cancer specimens with associated pathological, clinical, and molecular data was analyzed by immunohistochemistry to assess the role of TFAP2D.

**Results:**

TFAP2D expression was typically increased in prostate cancer as compared to adjacent non-neoplastic glands. TFAP2D staining was considered negative in 24.3% and positive in 75.7% of 13,545 interpretable cancers. TFAP2D staining was significantly linked to advanced tumor stage, high classical and quantitative Gleason grade, lymph node metastasis, and a positive surgical margin (*p* ≤ 0.0045). TFAP2D positivity was more common in ERG fusion positive (88.7%) than in ERG negative cancers (66.8%; *p* < 0.0001). Subset analyses in 3776 cancers with and 4722 cancers without *TMPRSS2:ERG* fusion revealed that associations with tumor phenotype and patient outcome were largely driven by the subset of ERG negative tumors. Multivariate analysis did not identify TFAP2D protein expression levels as a robust independent prognostic parameter. Positive TFAP2D immunostaining was significantly associated with 10 of 11 previously analyzed chromosomal deletions in ERG negative cancers (*p* ≤ 0.0244 each) indicating that elevated TFAP2D expression parallels genomic instability in prostate cancer.

**Conclusion:**

These data demonstrate that TFAP2D protein overexpression is linked to prostate cancer progression and genomic instability in ERG negative prostate cancers.

## Introduction

Prostate cancer is the most prevalent cancer in men in developed countries and is clinically characterized by a broad spectrum of tumor phenotype from incidentally discovered and clinically silent tumors to highly aggressive and metastasizing tumors with significant mortality (Bray et al. 2018). To predict tumor behavior, clinical parameters such as serum PSA-levels as well as histopathologic criteria, especially Gleason tumor grading, are widely used. However, these methods may lack reliable prediction of disease course in individual cases. To more reliably prevent unnecessary treatments better prognostic molecular markers are needed.

The transcription factor AP-2 family consists of five isoforms (AP-2α to AP-2ε) that modulate gene expression after dimerization via binding to palindromic GC-rich sequences in promotor and enhancer regions of various genes that impart cellular proliferation and differentiation (Eckert et al. 2005; Williams and Tjian 1991). An essential role in embryology/organ development, especially for neuronal/neuroectodermal tissue (AP2α, AP2β, AP2γ, AP2δ) but also in the kidney (AP2β, AP2γ), eye (Ap2δ) and olfactoric bulb (AP2ε) with varying redundancy between the isoforms was reported (Zhao et al. 2003; Moser et al. 1997a; Feng and Williams 2003; Moser et al. 1997b; Werling and Schorle 2002). The isoform AP-2δ (TAFP2D) is also expressed in adult tissue of the male genital tract, namely in the prostate and with less abundance in testicular tissue (Cheng et al. 2002).

Few reports have analyzed the role of AP-2 family members in tumorigenesis. In this context, most studies focus on breast cancer, where AP-2 responsive elements were found in the estrogen receptor gene (ESR1) and the cERBB2 gene (HER2/neu). Upregulation of AP-2 in cancer cell was detected, and tumor progression in a murine model of breast cancer with AP-2γ overexpression was reported (Turner et al. 1998; Bosher et al. 1996; Jager et al. 2005; Pellikainen et al. 2004). TFAP2G is also overexpressed in germ cell tumors (and its precursor in-situ lesion) of the testis, supporting the notion of oncofetal properties of AP-2 family members (Pauls et al. 2005).

Based on the reported role of TFAP2D in prostate tissue and the implications of AP-2 family members in neoplasia (Cheng et al. 2002), we aimed to determine the potential role of varying TFAP2D expression levels in prostate cancer. For this purpose, TFAP2D protein expression was successfully analyzed in 13,545 of 17,747 prostate cancers that were available in a tissue microarray format.

## Materials and methods

### Patients

Radical prostatectomy specimens were available from 17,747 patients, undergoing surgery between 1992 and 2015 at the Department of Urology and the Martini Clinic at the University Medical Center Hamburg-Eppendorf. Histological analysis was performed in standardized manner including complete embedding of the entire prostate (Schlomm et al. 2008). Histopathological data were retrieved from the patients’ records, including tumor stage, Gleason grade, nodal stage, and status of the resection margin. “Quantitative” Gleason grading was performed as described (Sauter et al. 2016). In brief, for every prostatectomy specimen, the percentages of Gleason 3, 4, and 5 patterns were recorded in cancerous tissues as part of the regular process of Gleason grading. Gleason 3 + 4 and 4 + 3 cancers were subdivided according to their percentage of Gleason 4. For practical use, cancers within the 3 + 4 and 4 + 3 categories were allocated into 8 subgroups: 3 + 4 ≤ 5% Gleason 4, 3 + 4 6–10%, 3 + 4 11–20%, 3 + 4 21–30%, 3 + 4 31–49%, 4 + 3 50–60%, 4 + 3 61–80% and 4 + 3 > 80% Gleason 4. Separate groups were defined for cancers with tertiary Gleason 5 pattern, including 3 + 4 Tert.5 and 4 + 3 Tert.5. From 14,464 patients follow-up data with a mean follow-up of 56.3 months was available (median 48; Table 1). Prostate specific antigen (PSA) values were measured subsequent to surgery. PSA recurrence was defined as the time point when postoperative PSA was at least 0.2 ng/ml and an PSA increase at subsequent measurements was observed. The TMA manufacturing process was described earlier in detail (Kononen et al. 1998). In short, a single 0.6 mm tissue core was taken from one donor tissue block of each patient. The donor block was merely selected for high tumor cell content, but not for a particular tumor focus or Gleason pattern in order to avoid a potential selection bias towards focal but potentially non-representative tumor areas. The tissues were distributed among 27 TMA blocks, each containing 144 to 522 tumor samples. For internal controls, each TMA block also contained various control tissues, including normal prostate tissue. The molecular database attached to the TMA contained previously compiled data on ERG expression in 10,678 (Weischenfeldt et al. 2013), ERG break-apart FISH analysis in 7099 (expanded from (Minner et al. 2011), Ki67-labeling index in 4426 (expanded from (Minner et al. 2010), androgen receptor (AR) expression in 7856 cancers (Weischenfeldt et al. 2013) and deletion status of 3p14 (FOXP1) in 7201 cases (expanded from (Krohn et al. 2013), 5q21 (CHD1) in 8074 (expanded from (Burkhardt et al. 2013), 6q15 (MAP 3 K7) in 6069 cases (expanded from (Kluth et al. 2013), 8p21 in 7001 cases (expanded from (Kluth et al. 2017), PTEN (10q23) in 6803 cases (expanded from (Krohn et al. 2012), 12p13 (CDKN1B) in 6187 cases (expanded from (Kluth et al. 2015a), 12q24 in 7435 cases (expanded from (Weischenfeldt et al. 2013), 13q14 in 7499 cases (expanded from (Kluth et al. 2018), 16q24 in 5493 cases (expanded from (Kluth et al. 2015b), 17p13 (TP53) in 8307 cases (expanded from (Kluth et al. 2014), and 18q21 in 7032 cases (expanded from (Kluth et al. 2016). The usage of archived diagnostic left-over tissues for manufacturing of tissue microarrays, their analysis for research purposes and patient data analysis has been approved by local laws (HmbKHG, §12,1) and by the local ethics committee (Ethics commission Hamburg, WF-049/09). All work has been carried out in compliance with the Helsinki Declaration.

**Table 1:** Composition of the prostate prognosis tissue microarray. Percentage in the column “Study cohort on TMA” refers to the fraction of samples across each category. Percentage in column “Biochemical relapse among categories” refers to the fraction of samples with biochemical relapse within each parameter in the different categories

	No. of patients (%)	
	Study cohort on TMA (*n* = 17,747)	Biochemical relapse among categories
Follow-up (mo)		
n	14,464 (81.5%)	3612 (25%)
Mean	56.3	–
Median	48	–
Age (y)		
≤ 50	433 (2.4%)	66 (15.2%)
51–59	4341 (24.5%)	839 (19.3%)
60–69	9977 (56.4%)	2073 (20.8%)
≥ 70	2936 (16.6%)	634 (21.6%)
Pretreatment PSA (ng/ml)		
< 4	2225 (12.6%)	313 (14.1%)
4–10	10,520 (59.6%)	1696 (16.1%)
10–20	3662 (20.8%)	1043 (28.5%)
> 20	1231 (7%)	545 (44.3%)
pT stage (AJCC 2002)		
pT2	11,518 (65.2%)	1212 (10.5%)
pT3a	3842 (21.7%)	1121 (29.2%)
pT3b	2233 (12.6%)	1213 (54.3%)
pT4	85 (0.5%)	63 (74.1%)
Gleason grade		
≤ 3 + 3	3570 (20.3%)	264 (7.4%)
3 + 4	9336 (53%)	1436 (15.4%)
3 + 4 Tert.5	798 (4.5%)	165 (20.7%)
4 + 3	1733 (9.8%)	683 (39.4%)
4 + 3 Tert.5	1187 (6.7%)	487 (41%)
≥ 4 + 4	999 (5.7%)	531 (53.2%)
pN stage		
pN0	10,636 (89.4%)	2243 (21.1%)
pN+	1255 (10.6%)	700 (55.8%)
Surgical margin		
Negative	14,297 (80.8%)	2307 (16.1%)
Positive	3388 (19.2%)	1304 (38.5%)

Numbers do not always add up to 17,747 in the different categories because of cases with missing data. *Abbreviation*: *AJCC* American Joint Committee on Cancer

### Immunohistochemistry

TMA sections were freshly cut and immunostained on 1 day and in one experiment. Slides were exposed to heat-induced antigen retrieval for 5 min in an autoclave at 121 °C in pH 7.8 Tris-EDTA buffer after deparaffinization. Primary antibody specific for Anti-TFAP2D (rabbit polyclonal antibody, Sigma-Aldrich, St. Louis, Missouri, USA, HPA048962; dilution 1:150) was applied at 37 °C for 60 min. Bound antibody was then visualized using the EnVision Kit (Dako, Glostrup, Denmark) according to the manufacturer’s directions. TFAP2D staining was mainly nuclear, however, often accompanied by weak to moderate cytoplasmic staining. Nuclear staining was scored in this study because of the known nuclear function of TFAP2D. As TFAP2D typically stained the nucleus in all (100%) tumor cells of a TFAP2D positive tissue spot, only the staining intensity was assessed in a four-step scale including negative, weak (1+), moderate (2+), and strong (3+) staining.

### Statistics

For statistical analysis JMP®12 software (SAS Institute Inc., NC, USA) was used. Chi^2^-test and contingency tables were performed to check for associations between molecular parameters and tumor phenotype. Cox proportional hazards regression analysis was performed to test the statistical independence and significance between pathological, molecular and clinical variables. Separate analyses were performed using different sets of parameters available either before or after prostatectomy. Survival curves were calculated according to Kaplan-Meier. The Log-Rank test was performed to find significant differences between groups.

## Results

### Technical issues

A total of 13,545 of 17,747 tumor samples (76.3%) were interpretable in our TMA analysis. Reasons for non-informative cases (*n* = 4202; 23.7%) included lack of tissue samples or absence of unequivocal cancer tissue in the TMA spot.

### TFAP2D expression in normal and cancerous prostate tissues

**Fig. 1:**
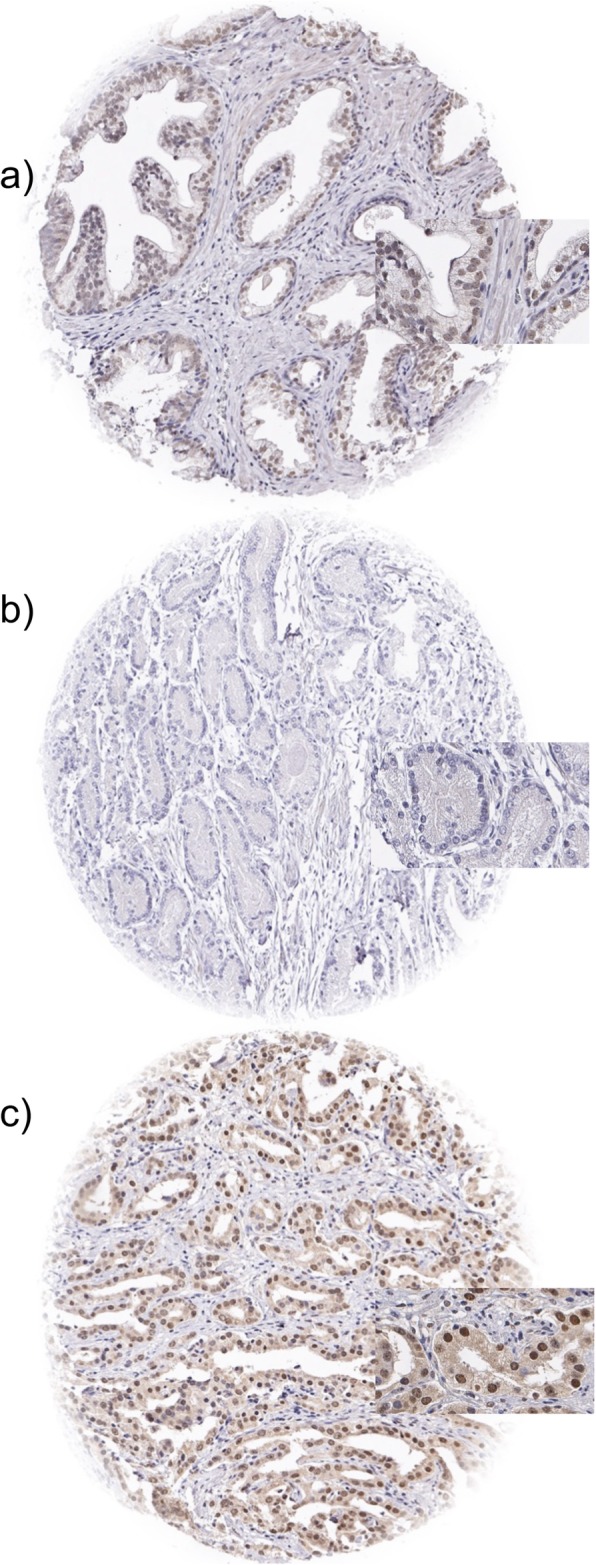
Examples of TFAP2D immunostainings in **a** normal prostate glands and cancer spots with **b** lack of staining and **c** nuclear staining

In normal prostate glands, nuclear TFAP2D staining intensity ranged from negative to moderate in luminal and basal cells. In prostate cancers, nuclear staining was seen in 10,259 of our 13,545 (75.7%) interpretable tumors. TFAP2D staining was considered weak in 73.4%, moderate in 2.3%, and strong in < 0.1% of cancers. Because of the low number of cases with moderate and strong staining (*n* = 313), tumors were classified as TFAP2D positive (any staining) and negative for all statistical analyses. Tissue spots containing both normal and cancerous glands usually showed higher TFAP2D levels in the tumor cells than in normal glands. Tumors with negative findings typically also lacked TFAP2D staining in the adjacent normal tissues. Representative micrographs depicting nuclear TFAP2D immunostaining are given in Fig. 1.

### TFAP2D expression and tumor phenotype

TFAP2D staining was significantly associated with adverse tumor features, including advanced tumor stage, high Gleason grade, presence of lymph node metastasis (*p* < 0.0001 each) and a positive surgical margin (*p* = 0.0045, Table 2).

**Table 2:** TFAP2D immunostaining results and prostate cancer phenotype in all cancers, ERG negative cancers, and ERG positive cancers

	TFAP2D IHC result all cancers				TFAP2D IHC result in ERG negative cancers				TFAP2D IHC result in ERG positive cancers			
	n 13545	negative (%) 24.3	positive (%) 75.7	*p* value	n 4722	negative (%) 33.2	positive (%) 66.8	*p* value	n 3776	negative (%) 11.3	positive (%) 88.7	*p* value
Tumor stage												
pT2	8569	26.6	73.4	< 0.0001	3114	35.3	64.7	< 0.0001	2268	11.3	88.7	0.8159
pT3a	3054	21.9	78.1		982	32.4	67.6		998	10.8	89.2	
pT3b-4	1866	17.1	82.9		612	23.2	76.8		495	11.9	88.1	
Gleason grade												
≤ 3 + 3	2391	31.8	68.2	< 0.0001	865	42.7	57.3	< 0.0001	725	14.3	85.7	0.0256
3 + 4	7246	24.7	75.3		2530	34	66		2203	10.9	89.1	
3 + 4 Tert.5	648	22.8	77.2		216	29.6	70.4		117	11.1	88.9	
4 + 3	1354	18.8	81.2		518	25.3	74.7		374	8.8	91.2	
4 + 3 Tert.5	988	15.9	84.1		305	23.9	76.1		211	7.6	92.4	
≥ 4 + 4	809	18.3	81.7		284	23.6	76.4		143	13.3	86.7	
Gleason grade quant												
3 + 4 ≤ 5%	1839	27.8	72.2	< 0.0001	673	37.7	62.3	< 0.0001	550	12.7	87.3	0.0636
3 + 4 6–10%	1811	25.1	74.9		663	34.5	65.5		582	10.3	89.7	
3 + 4 11–20%	1609	23.8	76.2		574	34.3	65.7		488	9.4	90.6	
3 + 4 21–30%	825	21.6	78.4		288	29.2	70.8		284	9.2	90.8	
3 + 4 31–49%	682	22.1	77.9		256	30.9	69.1		209	12	88	
4 + 3 50–60%	564	19.5	80.5		216	26.9	73.1		168	8.3	91.7	
4 + 3 61–80%	495	17.6	82.4		206	23.8	76.2		143	7.7	92.3	
4 + 3 > 80%	125	16	84		52	19.2	80.8		33	12.1	87.9	
Lymph node metastasis												
N0	8141	23.8	76.2	< 0.0001	2725	32.6	67.4	< 0.0001	2142	11.4	88.6	0.4192
N+	1032	15.6	84.4		285	20.7	79.3		242	13.2	86.8	
Preop. PSA level (ng/ml)												
< 4	1624	21.6	78.4	0.0101	487	30.4	69.6	0.2017	526	10.6	89.4	0.012
4–10	7988	24.2	75.8		2797	33.6	66.4		2332	10.2	89.8	
10–20	2846	24.8	75.2		1033	31.8	68.2		661	14.1	85.9	
> 20	1008	27.1	72.9		384	36.5	63.5		231	15.2	84.8	
Surgical margin												
negative	10,764	24.8	75.2	0.0045	3743	33.6	66.4	0.2278	2983	11.5	88.5	0.4099
positive	2732	22.2	77.8		967	31.5	68.5		777	10.4	89.6	

### TFAP2D and *TMPRSS2:ERG* fusion status

**Fig. 2:**
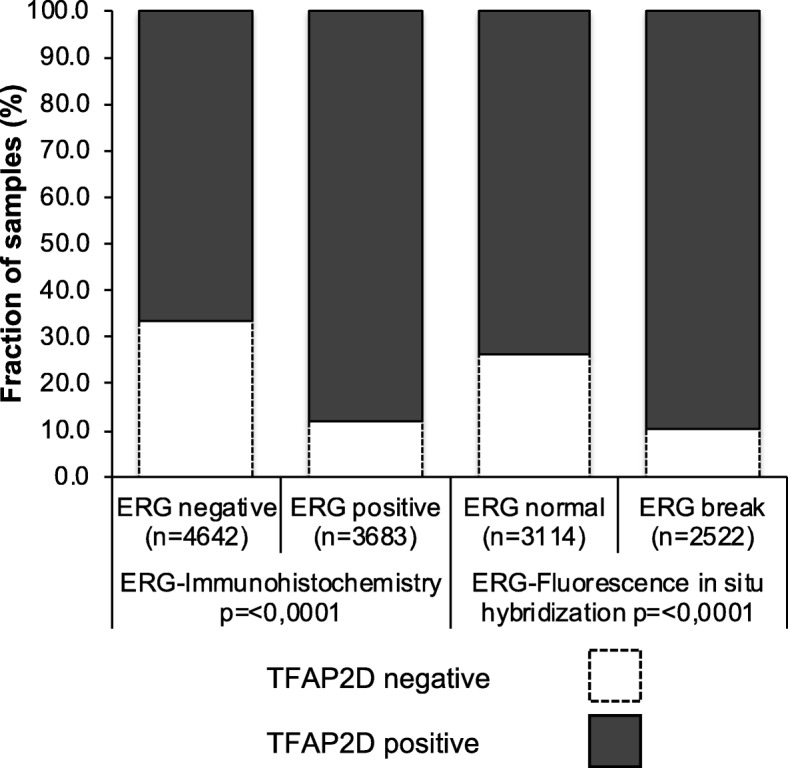
Association between positive TFAP2D immunostaining and ERG-status (IHC/FISH) in all cancers

**Fig. 3:**
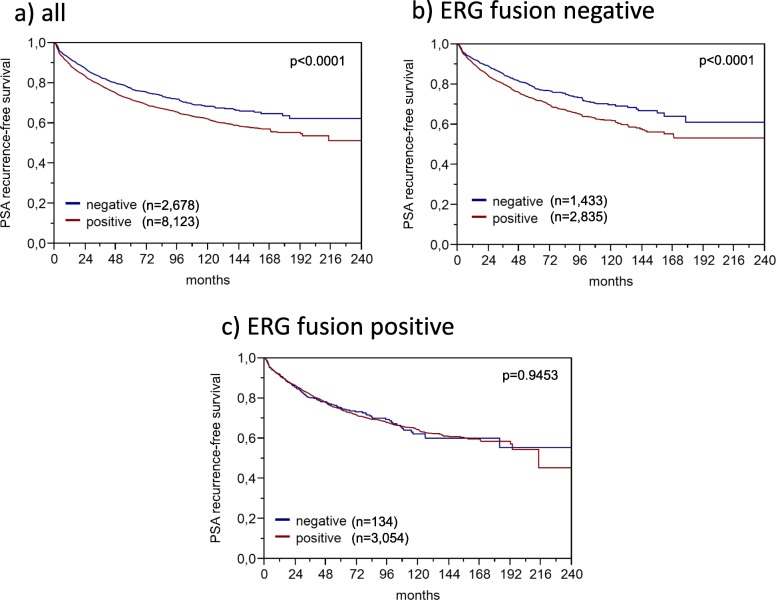
Kaplan-Meier plot of prostate specific antigen (PSA) recurrence-free survival after radical prostatectomy and immunostaining of TFAP2D in **a** all cancers, **b** the ERG negative, and **c** the ERG positive cancers

Data on *TMPRSS2:ERG* fusion status obtained by FISH were available from 5636 and by immunohistochemistry from 8325 tumors with evaluable TFAP2D immunostaining. A concordant result of IHC and FISH determined ERG status was found in 5291 of 5535 (95.6%) cancers for which both data were available. High TFAP2D expression was strongly linked to *TMPRSS2:ERG* rearrangement and ERG expression: TFAP2D positivity increased from 66.5–73.5% in ERG negative cancers (by IHC or FISH) to 88.2–89.8% in ERG positive cancers (*p* < 0.0001 each, Fig. 2). Because of the strong link between increased TFAP2D levels and ERG rearrangement, the impact of TFAP2D expression on tumor phenotype and prognosis was separately analyzed in ERG fusion positive and negative cancers. This analysis revealed that the observed associations were largely caused by the subset of ERG negative cancers, while TFAP2D staining was unrelated to the analyzed features in ERG positive cancers (Table 2). This especially held true for associations with patient outcome. Detectable TFAP2D expression was strongly linked to outcome in ERG negative cancers (*p* < 0.0001, Fig. 3b) but completely unrelated to patient outcome in ERG positive cancers (*p* = 0.9543, Fig. 3c).

### TFAP2D and chromosomal deletions

**Fig. 4:**
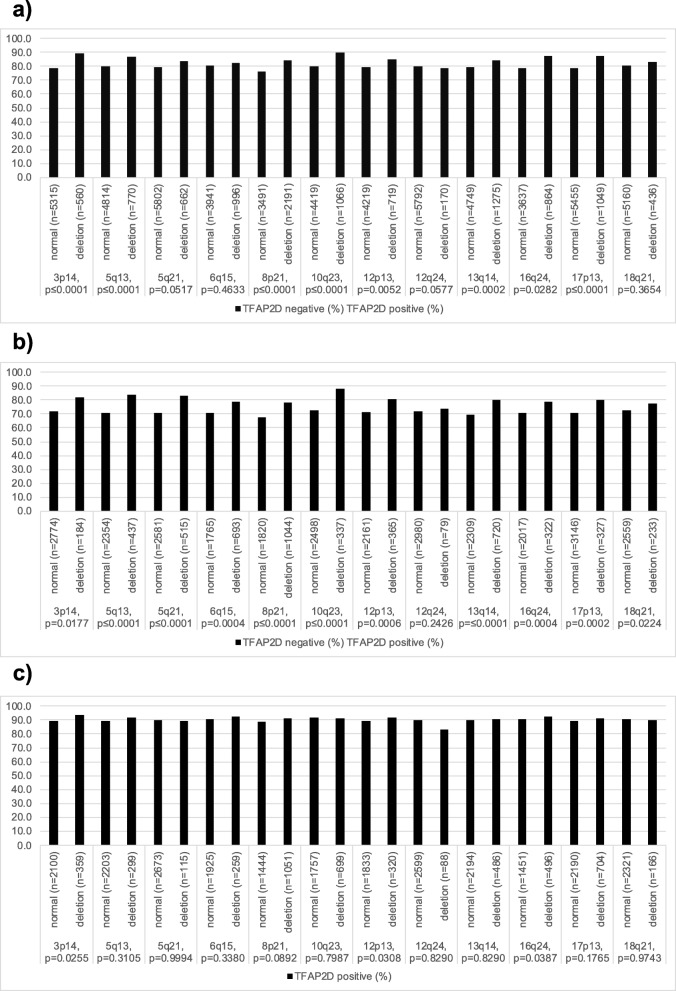
TFAP2D immunostaining and common genetic deletions in **a** all cancers, **b** ERG-negative cancers, and **c** ERG-positive cancers

Chromosomal deletions were generally more frequent in TFAP2D positive than negative cancers (Fig. 4). This observation was particularly strong in ERG negative cancers, where it reached statistical significance in 10 out of 11 deletions (Fig. 4b). This association was much less strong in ERG positive cancers where it only reached statistical significance for 3p14, and 12p13, and 16q24 (Fig. 4c).

### TFAP2D, tumor cell proliferation (Ki67 labeling index) and androgen receptor (AR) expression

**Fig. 5:**
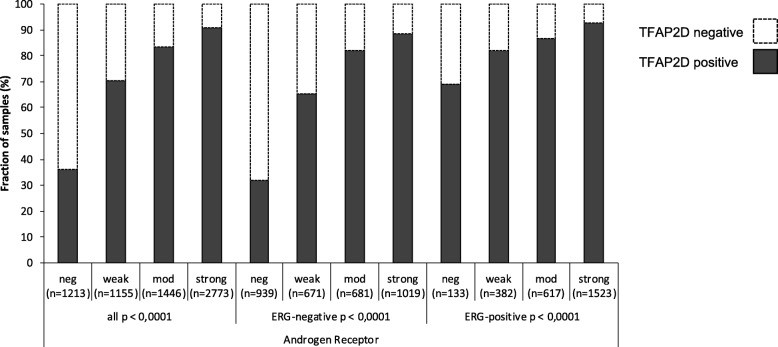
Association between positive TFAP2D immunostaining and androgen receptor (AR) status in all cancers, ERG negative and ERG positive cancers

TFAP2D staining was significantly linked to increased cell proliferation as determined by Ki67 labeling index (Table 3). This association was independent of Gleason grading (*p* < 0.0001) and also held true for most analyzed subgroups with identical Gleason score (*p* ≤ 0.0025 each). There was a strong positive association between AR expression and presence of nuclear TFAP2D staining (*p* < 0.0001, Fig. 5). Whereas nuclear TFAP2D staining was observed in 35.9% of tumors with negative AR expression, 90.9% of tumors with strong AR expression were TFAP2D positive. The observed association held true for both ERG positive and ERG negative subgroups and was particularly evident in the ERG negative subgroup (*p* < 0.0001 each).

**Table 3:** TFAP2D immunostaining and Ki67 labeling index

ki67	TFAP2D	*n*=	Ki67 Li (mean)		standard error of the mean
all *p* < 0.0001	negative	1376	1.89	±	0.07
	positive	4328	3.08	±	0.04
Gleason ≤3 + 3 *p* < 0.0001	negative	357	1.55	±	0.11
	positive	808	2.51	±	0.07
Gleason 3 + 4 *p* < 0.0001	negative	730	1.81	±	0.09
	positive	2483	2.91	±	0.05
Gleason 3 + 4 Tert.5 *p* < 0.0001	negative	61	2.10	±	0.32
	positive	172	3.59	±	0.19
Gleason 4 + 3 *p* = 0.0025	negative	112	2.44	±	0.30
	positive	435	3.45	±	0.15
Gleason 4 + 3 Tert.5 *p* < 0.0001	negative	59	2.12	±	0.49
	positive	233	4.33	±	0.25
Gleason ≥4 + 4 *p* = 0.0504	negative	56	3.57	±	0.61
	positive	194	4.94	±	0.33

### TFAP2D expression and PSA recurrence

Follow-up data were available from 10,801 patients with interpretable TFAP2D immunostaining. Nuclear TFAP2D staining was linked to early biochemical recurrence (*p* < 0.0001, Fig. 3a). Subset analyses of ERG positive and ERG negative cancers revealed that the prognostic impact of TFAP2D expression was driven by the ERG-negative group (*p* < 0.0001, Fig. 3b). TFAP2D expression was unrelated to patient outcome in ERG-positive cancers (*p* = 0.9453, Fig. 3c). A further analysis based on subsets of all cancers with identical classical and quantitative Gleason grades revealed no significant prognostic impact of TFAP2D expression for any Gleason group (Additional Figure 1).

### Multivariate analysis

Four different multivariate analyses were performed to investigate the clinical relevance of TFAP2D immunostaining in different scenarios. Scenario 1 evaluated all postoperatively available prognostic parameters including pT, pN, surgical margin status, preoperative PSA value and prostatectomy Gleason grade. In scenario 2, the same postoperatively available parameters were included with the exception of pN. This was because the indication and extent of lymph node dissection is not standardized in the surgical therapy of prostate cancer, which may introduce a bias towards high grade cancers. The next two scenarios were to model the preoperative situation to the best possible extent. Scenario 3 included TFAP2D immunostaining, preoperative serum PSA, clinical tumor stage (cT) and the prostatectomy Gleason grade. Since postoperative determination of the Gleason grade is superior to the preoperative biopsy Gleason grade (subjected to sampling errors and under-grading in more than one third of cases (Epstein et al. 2012)), this parameter was replaced by the original preoperative biopsy Gleason grade in Scenario 4. The results of this analysis show that an independent prognostic role of TFAP2D measurement was limited to the pre-surgical scenario 4 in all cancers and ERG negative cancers (Table 4, *p* = 0.0007 each).

**Table 4:** Multivariate Cox regression analysis including established prognostic parameters and the TFAP2D expression in all prostate cancers and in the subsets of ERG negative and ERG positive prostate cancers

Tumor subset	Scenario	n	*p*-value							
			preoperative PSA-Level	pT Stage	cT Stage	Gleason grade prostatectomy	Gleason grade biopsy	pN stage	R status	TFAP2D Expression
all cancers	1	10,827	<.0001*	<.0001*	–	<.0001*	–	<.0001*	<.0001*	0.2379
	2	10,846	<.0001*	<.0001*	–	<.0001*	–	–	<.0001*	0.2712
	3	10,692	<.0001*	–	<.0001*	<.0001*	–	–	–	0.0496*
	4	9204	<.0001*	–	<.0001*	–	<.0001*	–	–	0.0007*
ERG-negative cancers	1	4327	<.0001*	<.0001*	–	<.0001*	–	0.8751	<.0001*	0.4114
	2	4334	<.0001*	<.0001*	–	<.0001*	–	–	0.0040*	0.4285
	3	4298	<.0001*	–	<.0001*	<.0001*	–	–	–	0.1722
	4	4228	<.0001*	–	<.0001*	–	<.0001*	–	–	0.0007*
ERG- postive cancers	1	3429	<.0001*	<.0001*	–	<.0001*	–	<.0001*	0.0001*	0.61
	2	3437	<.0001*	<.0001*	–	<.0001*	–	–	<.0001*	0.822
	3	3383	<.0001*	–	<.0001*	<.0001*	–	–	–	0.8684
	4	3327	<.0001*	–	<.0001*	–	<.0001*	–	–	0.2301

Scenario 1 includes all postoperatively available parameters (pathological tumor (pT) stage, lymph node (pN), surgical margin (R) status, preoperative PSA value and Gleason grade obtained after the morphological evaluation of the entire resected prostate. Scenario 2 excluded the nodal status from analysis. Scenario 3 included preoperative PSA, clinical tumor (cT) stage and Gleason grade obtained on the prostatectomy specimen. In scenario 4, the preoperative Gleason grade obtained on the original biopsy was combined with preoperative PSA, and cT stage (* = significant)

## Discussion

The results of our study demonstrate that nuclear TFAP2D protein expression is a predictor of poor prognosis in ERG negative prostate cancer.

Nuclear TFAP2D staining was seen in 75.7% of 13,545 interpretable prostate cancers whereas adjacent normal prostatic epithelial cells were only occasionally TFAP2D positive. This suggests TFAP2D to be overexpressed during prostate cancer development. Published immunohistochemical studies on TFAP2D are currently lacking. Available RNA expression data from The Cancer Genome Atlas (TCGA) shown on the Human Protein Atlas website (https://www.proteinatlas.org/ENSG00000008197-TFAP2D/pathology) do currently not support a prognostic role of TFAP2D mRNA expression. However, several other AP-2 family members have been reported to be differentially expressed in tumors. These include studies in prostate cancer where AP-2α was described to be downregulated in prostate cancer as compared to non-tumorous tissue by Ruiz et al. (Ruiz et al. 2001). AP-2γ levels are increased in breast cancer cells in contrast to adjacent non-tumorous tissue (Turner et al. 1998; Pellikainen et al. 2004) and germ cell tumors show strong AP-2γ staining in contrast to non-neoplastic testicular tissue (Pauls et al. 2005; Hoei-Hansen et al. 2004). A role of AP-2 family members in cancer development has also been supported by functional studies showing oncogenic activity and interaction with important cancer pathways. Overexpression of AP-2γ promotes tumorigenesis in a murine breast cancer model, suggesting an oncogenic role of AP2γ in breast cancer (Jager et al. 2005). AP-2α and AP-2β regulate cKIT through an AP-2 binding site in a tumor suppressive manner in melanoma cell lines (Huang et al. 1998). AP-2α can confer tumor-suppressive properties via enhancing p53-mediated transcriptional activity (McPherson et al. 2002). The significant association of elevated TFAP2D expression levels with unfavorable prostate cancer phenotype and prognosis supports an in vivo role of TFAP2D in prostate cancer progression. Lipponen et al. earlier demonstrated increased nuclear expression of AP-2α to be associated with aggressive tumor phenotype in prostate cancer (Lipponen et al. 2000).

TFAP2D is not an extensively studied protein. The molecular database collected through earlier studies on the same patient cohort enabled us to study the in vivo relationship between TFAP2D expression and molecular parameters of interest. For this study, we had selected androgen receptor protein expression because of its pivotal role in prostate cancer, *TMPRSS2:ERG* fusion because this is the most common molecular alteration in prostate cancer, 11 different chromosomal deletions because these represent the most common recurrent genomic alterations in prostate cancer after *TMPRSS2:ERG* fusions, and tumor cell proliferation (Ki-67 labeling index). *TMPRSS2:ERG* fusions affect about 50% of prostate cancers (Brase et al. 2011; Tomlins et al. 2008) and lead to a constitutive overexpression of the transcription factor ERG. ERG expression completely lacks prognostic relevance (Minner et al. 2011). However, ERG modulates the expression of more than 1600 genes in prostate epithelial cells. The biological effects of various proteins may be mitigated or intensified in such a modified cellular microenvironment. The increased frequency of TFAP2D positive cancers in ERG positive (90%) compared to ERG negative subsets (74%) suggests an interaction between ERG and TFAP2D, either directly or via modulation of shared common downstream targets. The Wnt signaling cascade, whose ERG-dependent activation has extensively been analyzed (Brase et al. 2011; Wu et al. 2013; Li et al. 2011) may connect the TMPRSS2:ERG fusion status to TFAP2D expression. In a study on human colorectal cancer cell lines the Wnt pathway was found to be affected by AP2 family members in a direct manner via protein interaction (Li and Dashwood 2004).

The strong association of TFAP2D with androgen receptor expression is consistent with previous reports describing hormone-receptor mediated effects of other AP-2 family members. For instance, AP-2 binding sites are present in the promotor of the estrogen receptor which has implications for breast and endometrial cancer (Lin et al. 2016; Woodfield et al. 2009). A direct effect of TFAP2D via AP-2 binding sites within the androgen receptor gene is not known, however the epigenetic regulator protein EZH2 may link androgen-dependent and TFAP2D pathways. Beyond silencing gene expression via its histone methyltransferase activity, EZH2 can directly coactivate the androgen receptor and other transcription factors in prostate cancer (Xu et al. 2012). The promotor region of TFAP2D harbors EZH2 binding sites (Fishilevich et al. 2017). As cellular proliferation is dependent on the androgen receptor function in prostate cancer, it is possible, that the significant link between elevated Ki67 labeling index and high TFAP2D expression is also androgen receptor driven.

Our analysis of molecularly defined tumor subgroups revealed that the prognostic impact of TFAP2D expression was almost entirely driven by ERG negative cases. That an independent prognostic role of TFAP2D was limited to the pre-surgical situation underscores the prognostic power of classical post-surgical parameters such as the Gleason score that are difficult to beat for a molecular marker. An ERG specific cellular microenvironment may be responsible for the particularly prognostic role of TFAP2D-expression in ERG negative cancers or the mitigation of it in ERG negative cancers. It is not uncommon that the prognostic value of molecular features are limited to ERG positive (Burdelski et al. 2015; Burdelski et al. 2016a; Melling et al. 2015) or ERG negative cancers (Heumann et al. 2017; Burdelski et al. 2016b; Heumann et al. 2018). It should be kept in mind that the ERG dependent differences in prognostic value could be caused by the experimental set-up. The number of TFAP2D negative cases was rather low in the ERG positive subgroup (*n* = 134) for PSA recurrence. It cannot be excluded that the immunohistochemistry protocol developed for this project was better suited to distinguish expression differences in cancers with somewhat lower expression levels such as in ERG negative cancers than in tumors with higher expression, such as in ERG positive cancers. Irrespective of the reason behind, the selective prognostic impact of TFAP2D in ERG negative cancers demonstrates that prognostic markers (or their defining thresholds) depend on other molecular tumor features and the intracellular microenvironment of cancer cells. This represents a challenge for the development of prognostic cancer tests that shall be applicable to every patient.

Most chromosomal deletions occurring in prostate cancer are linked to either positive (*PTEN*, 3p, 8p, 16q, 17p) (Krohn et al. 2013; Kluth et al. 2017; Krohn et al. 2012; Kluth et al. 2015b; Kluth et al. 2014) or negative (6q, 5q, 13q, 18q) (Burkhardt et al. 2013; Kluth et al. 2013; Kluth et al. 2018; Kluth et al. 2016) ERG status. The evaluation whether a relationship exists between deletions and the expression of proteins that are also ERG related must therefore be done in subgroups of ERG positive and ERG negative cancers. That elevated TFAP2D expression was significantly associated with the majority of the analyzed deletions in ERG negative cancers highlights that elevated TFAP2D levels are either a cause or a consequence of genomic instability in prostate cancer cells. The transcriptional program of TFAP2D indeed affects genes with a role in DNA repair such as MMS19, a key player of nucleotide excision repair (Sun et al. 2007). That the association between TFAP2D expression levels and chromosomal deletions was visible in ERG negative but not in ERG positive cancers could again be explained by a biological role of the ERG related intracellular microenvironment on TFAP2D function or by issues related to the experimental set-up.

Moreover, the complete lack of a tendency towards a different outcome between TFAP2D positive and negative cancers defined by a specific classical or quantitative Gleason grade demonstrates the power of traditional morphologic parameters if it comes to predicting patient outcome. This represents another considerable challenge for the development of molecular prognostic parameters. It is noteworthy, however, that the Gleason Grade suffers from substantial interobserver variability reaching up to 40% in individual biopsies (Sauter et al. 2016). It is thus desirable not only to find independent but also better reproducible prognostic markers as compared to established parameters.

Some limitations are connected to our study. First, only one 0.6 mm tissue spot has been analyzed per cancer. Because prostate cancer is typically multifocal and heterogeneous, it cannot be excluded that the 75% TFAP2D positivity still underestimate the real frequency. However, using TMAs with a single spot per cancer, we have been able to reproduce a multitude of established associations between clinical features and molecular markers such as HER2 (Barlund et al. 2000), Vimentin (Moch et al. 1999) and Ki67 (Ruiz et al. 2006) in the past. Second, only one pathologist analyzed the TMA. We do not consider this as a serious drawback of our study. This is based on our finding that the experimental conditions in IHC studies have a much higher impact on the study outcome than for interobserver variability. For example, we have previously shown that the fraction of cancers staining positive for p53 can vary between 2.5 and > 90% if an “oversensitive” staining protocol was used (Schlomm et al. 2008). Third, we did not study TFAP2D in presurgical core needle biopsies but compared post prostatectomy IHC findings with pre-surgical clinical and histological parameters in our multivariate analysis. It is of note that the small amount of tissue in a 0.6 mm spot resembles that of core needle biopsies, making our TMA approach a suitable model for punch biopsy analysis.

## Conclusions

Upregulation of TFAP2D parallels genomic instability in prostate cancer and is associated with adverse tumor features, rapid cell proliferation and poor patient prognosis. If TFAP2D expression analysis will have a role for prostate cancer prognosis assessment, this will most likely be in combination with other biomarkers.

## Supplementary information

**Additional file 1: Figure S1.** Prognostic impact of TFAP2D expression in all cancers defined by the Gleason score. a) Impact of negative and positive TFAP2D expression as compared to the classical Gleason score categories. b-h) Impact of negative and positive TFAP2D expression as compared to the quantitative Gleason score categories defined by subsets of cancers with b) ≤5% Gleason 4 patterns, c) 6–10% Gleason 4 patterns, d) 11–20% Gleason 4 patterns, e) 21–30% Gleason 4 patterns, f) 31–49% Gleason 4 patterns, g) 50–60% Gleason 4 patterns, h) ≥61% Gleason 4 patterns.

## Data Availability

All data generated or analyzed during this study are included in this published article [and its supplementary information files].

## Abbreviations

AR: Androgen receptor
ERG: ETS-related gene
ETS: E26 transformation-specific
EZH2: Enhancer of zeste homolog 2
PSA: Prostate specific antigen
PTEN: Phosphatase and Tensin homolog
TFAP2D: Transcriptions factor AP-2δ
TMA: Tissue microarray
TMPRSS2: Transmembrane protease, serine 2
UICC: International Union Against Cancer
