# Supplementary figures and images for: Upregulation of the transcription factor TFAP2D is associated with aggressive tumor phenotype in prostate cancer lacking the TMPRSS2:ERG fusion
Source: Mol Med. 2020 Mar 6;26:24. doi: 10.1186/s10020-020-00148-4 (PMC7060561; doi:10.1186/s10020-020-00148-4)

## Slide 1
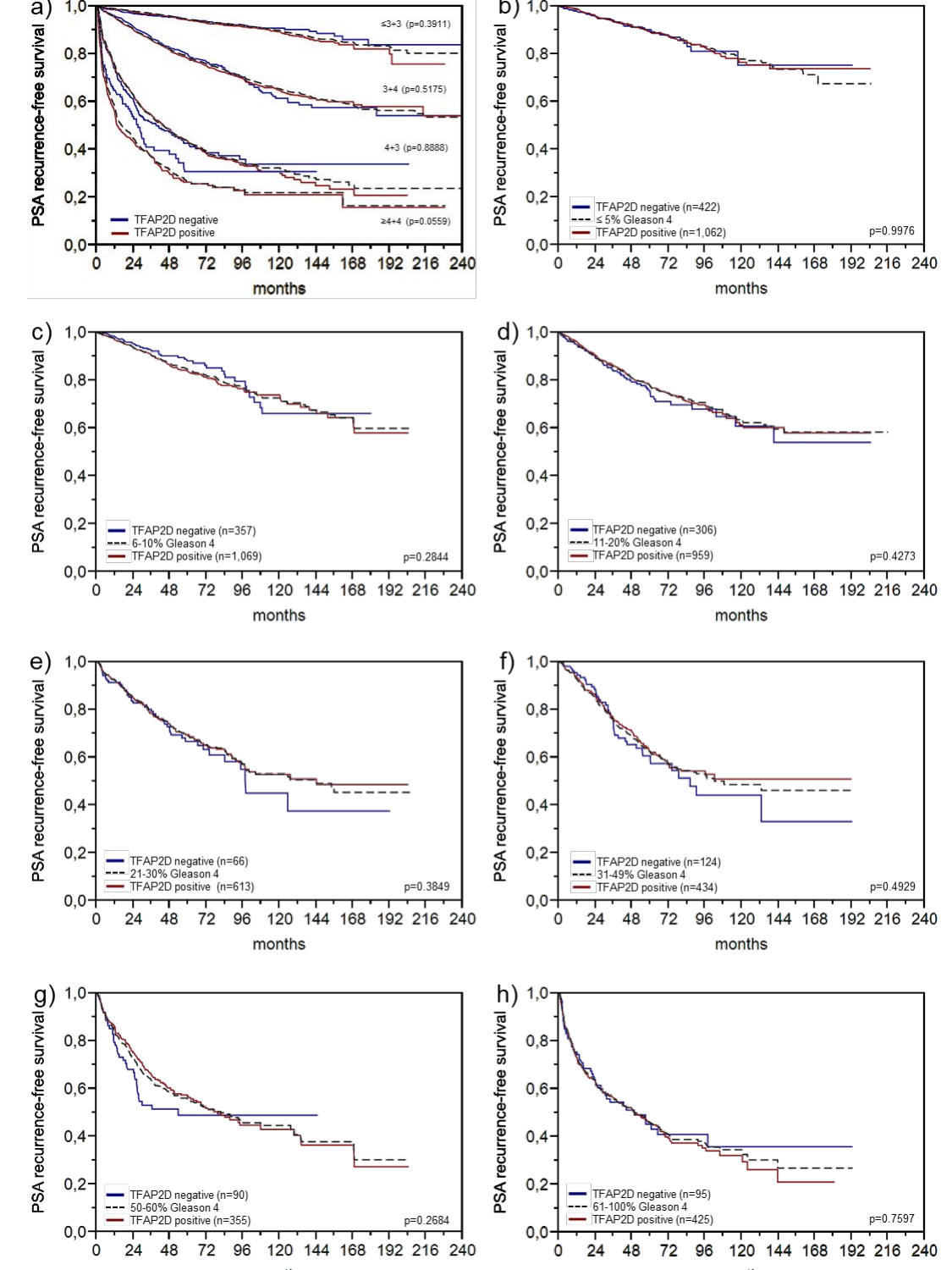

Supplement: Supplementary file 1 — Additional file 1: Figure S1. Prognostic impact of TFAP2D expression in all cancers defined by the Gleason score. a) Impact of negative and positive TFAP2D expression as compared to the classical Gleason score categories. b-h) Impact of negative and positive TFAP2D expression as compared to the quantitative Gleason score categories defined by subsets of cancers with b) ≤5% Gleason 4 patterns, c) 6–10% Gleason 4 patterns, d) 11–20% Gleason 4 patterns, e) 21–30% Gleason 4 patterns, f) 31–49% Gleason 4 patterns, g) 50–60% Gleason 4 patterns, h) ≥61% Gleason 4 patterns. [file 10020_2020_148_MOESM1_ESM.pptx]
